# Supplementary material for: Immune cell quantification of in situ inflammation partitions human lupus nephritis into mechanistic subtypes
Source: J Clin Invest. 2025 Sep 4;135(21):e192669. doi: 10.1172/JCI192669 (PMC12578395; doi:10.1172/JCI192669)
Supplement: Supplemental data [file jci-135-192669-s375.pdf]

A

ISN/RPS classification	(n)		
II	2		
III	3		
IV	11		
V	7		
Unknown	2		
**Age at SLE diagnosis**			(n)
Age, years (mean ± σ)	25.75	±16.0	12
Age range, years	10-61		12
**Age at biopsy**			(n)
Age, years (mean ± σ)	28.44	± 14.31	12
Age range, years	10-61		12

B

Banff subtype	(n)
IA	13
IB	7
Unknown	3

Donor type	(n)
Deceased	15
Living, related	1
Living, unrelated	2
Unknown	5

Allograft rejection subtype			(n)
T-cell mediated rejection			10
Mixed rejection			13
**Age at transplant**			(n)
Age, years (mean ± σ)	40.5	±19.5	23
Age range, years	10-72		23
**Age at biopsy**			(n)
Age, years (mean ± σ)	43.3	±18.5	23
Age range, years	15-73		23

C

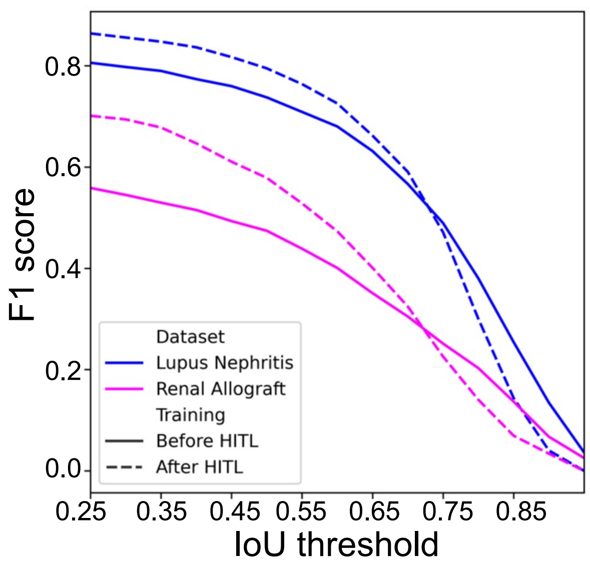

D

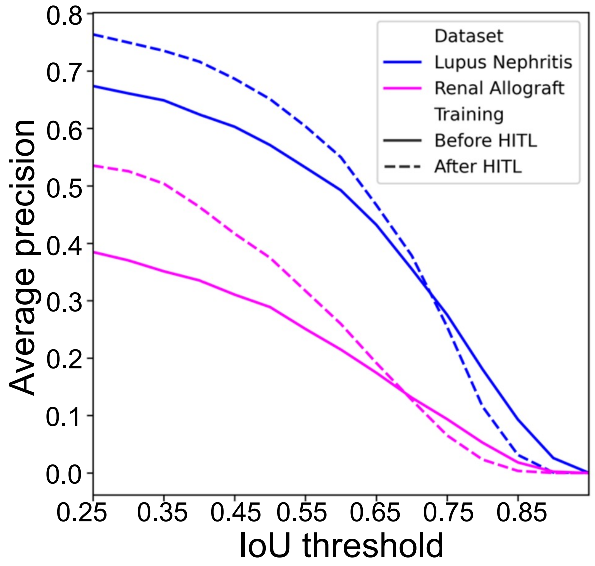

Casella, et. al, Supplemental Fig. 1

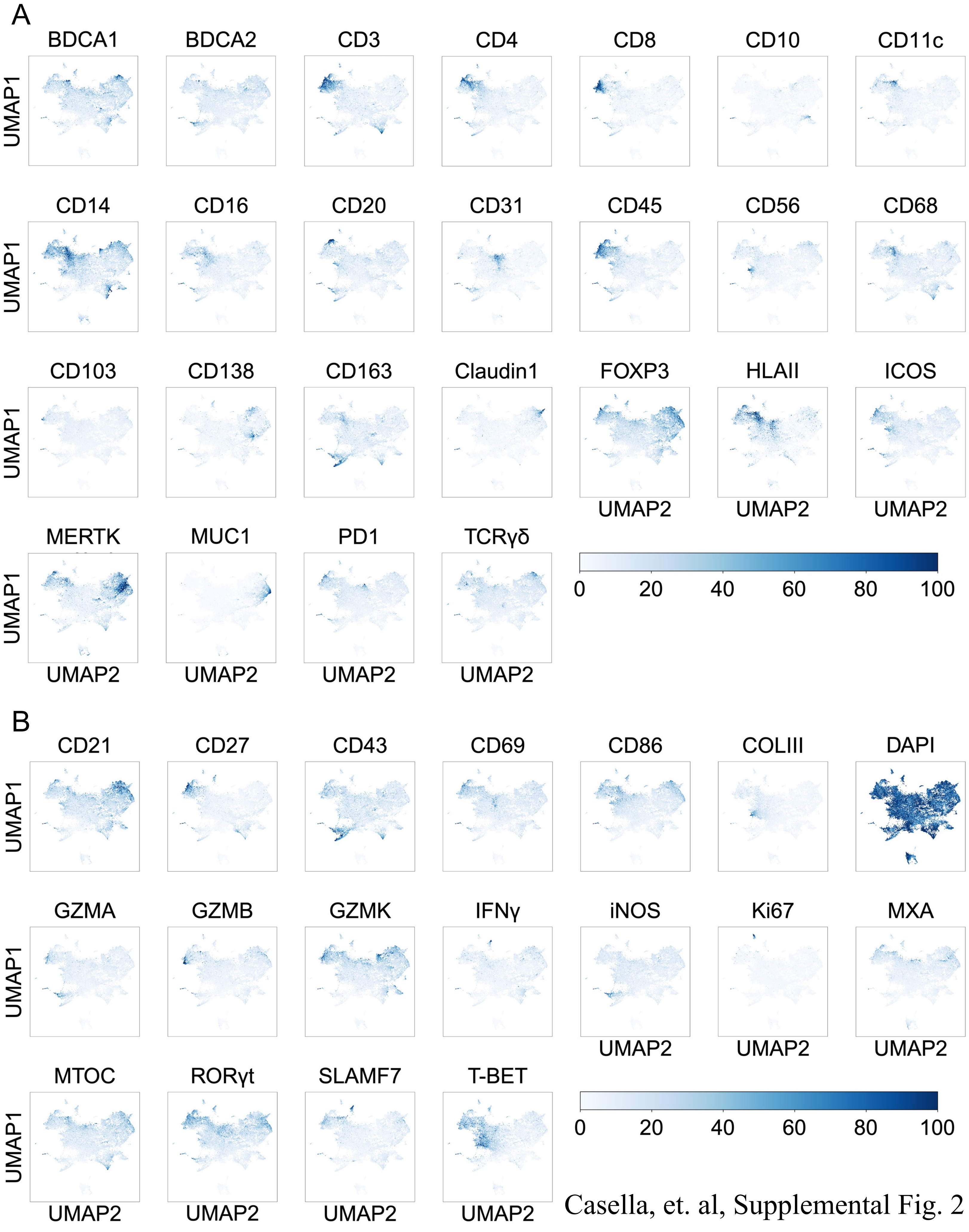

Casella, et. al, Supplemental Fig. 2

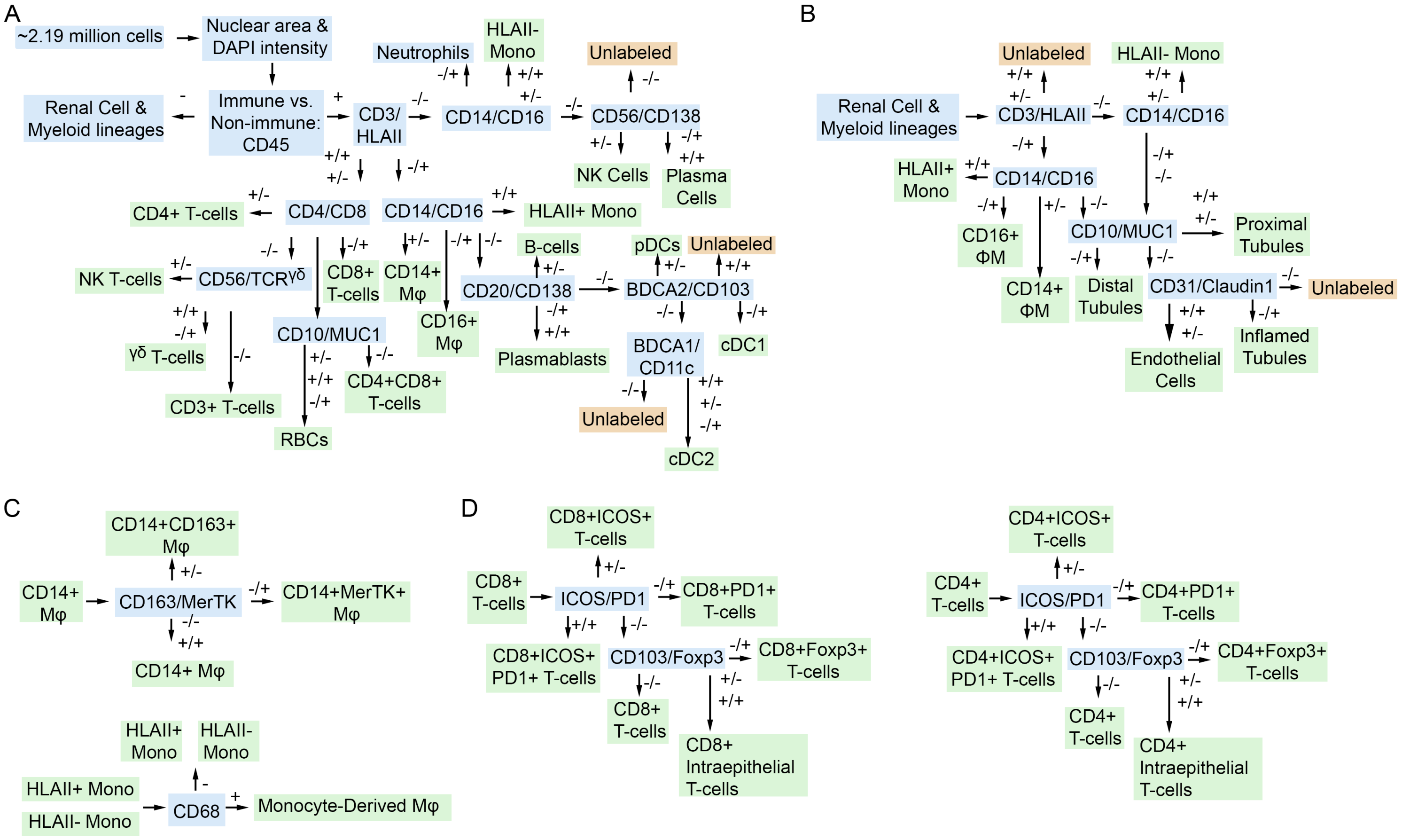

Casella, et. al, Supplemental Fig. 3

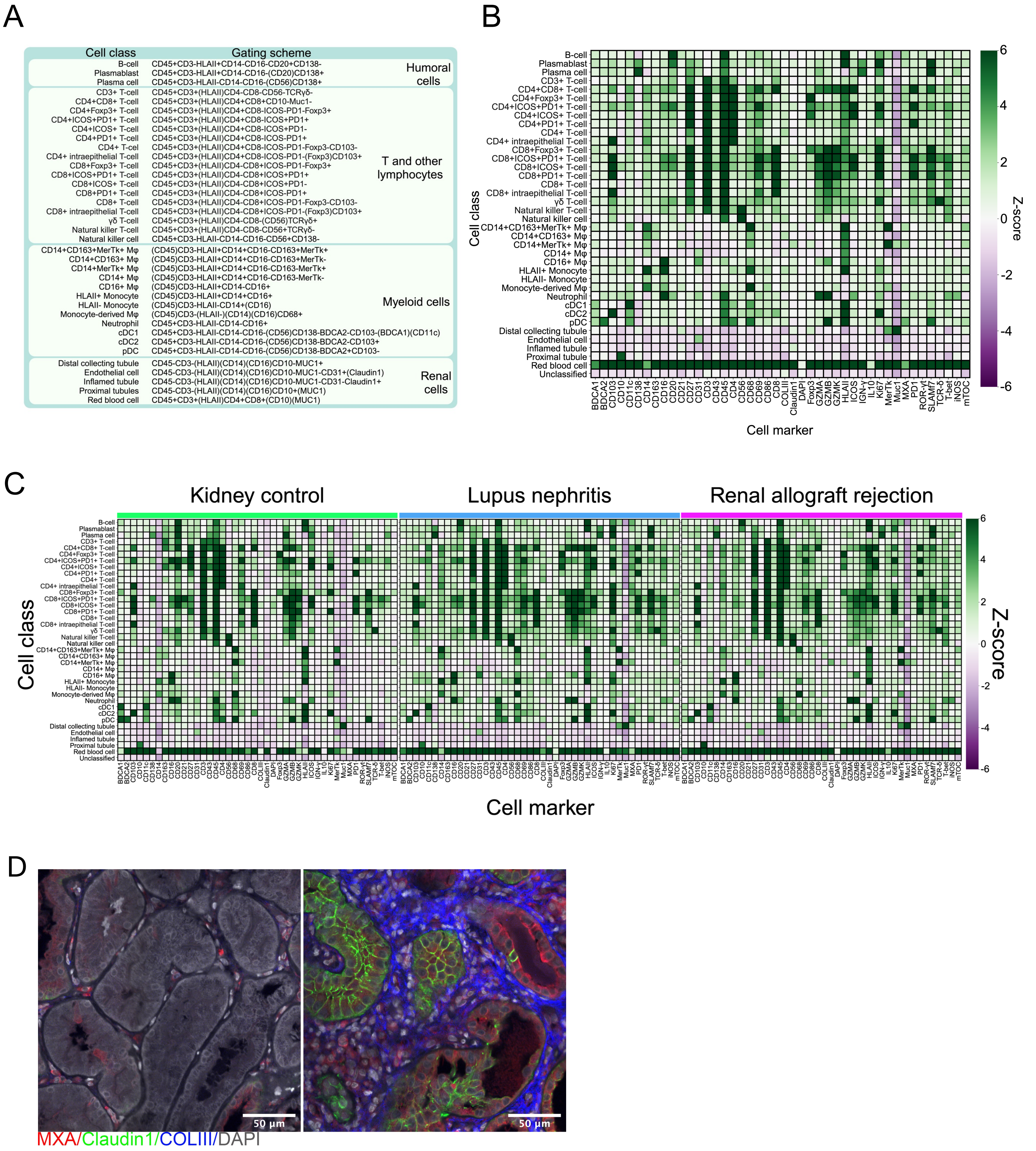

Casella, et. al, Supplemental Fig. 4

KC

Cell class	Count	Percentage
General unclassified	250481	36.74
Proximal tubules	136016	19.95
Endothelial cells	79185	11.62
Inflamed tubules	77049	11.3
Distal collecting tubules	72926	10.7
CD3+ T-cells	12233	1.79
CD14+ Mφs	9058	1.33
HLAII- monocytes	7553	1.11
CD14+CD163+ Mφs	7076	1.04
HLAII+ monocytes	4437	0.65
CD8+ T-cells	3713	0.54
Neutrophils	3094	0.45
Plasma cells	3033	0.44
CD16+ Mφs	2927	0.43
CD4+ T-cells	2535	0.37
Plasmablasts	1906	0.28
CD8+ intraepithelial T-cells	1719	0.25
CD4+ICOS+ T-cells	818	0.12
Monocyte-derived Mφs	776	0.11
TCRγδ T-cells	682	0.1
NK cells	613	0.09
cDC1s	478	0.07
CD14+MERTK+ Mφs	392	0.06
CD4+CD8+ T-cells	372	0.05
NK T-cells	321	0.05
B-cells	310	0.05
CD4+PD1+ T-cells	309	0.05
RBCs	299	0.04
cDC2s	293	0.04
CD14+CD163+MERTK+ Mφs	254	0.04
CD4+ICOS+PD1+ T-cells	223	0.03
CD4+Foxp3+ T-cells	216	0.03
CD8+ICOS+ T-cells	101	0.01
CD8+PD1+ T-cells	96	0.01
CD8+Foxp3+ T-cells	57	0.01
pDCs	45	0.01
CD4+ intraepithelial T-cells	43	0.01
CD8+ICOS+PD1+ T-cells	28	4.00E-03

LuN

Class_label	Count	Percentage
General unclassified	187499	23.91
Inflamed tubules	138193	17.62
Proximal tubules	109330	13.94
Endothelial cells	87288	11.13
Distal collecting tubules	66103	8.43
CD14+ Mφs	34871	4.45
CD14+CD163+ Mφs	32581	4.16
HLAII- monocytes	24784	3.16
HLAII+ monocytes	15377	1.96
CD3+ T-cells	13740	1.75
CD8+ T-cells	9868	1.26
CD14+CD163+MERTK+ Mφs	9519	1.21
CD14+MERTK+ Mφs	8800	1.12
Monocyte-derived Mφs	5829	0.74
CD4+ T-cells	5672	0.72
CD4+ICOS+PD1+ T-cells	3552	0.45
TCRγδ T-cells	3086	0.39
CD16+ Mφs	3069	0.39
CD4+ICOS+ T-cells	2825	0.36
RBCs	2672	0.34
Plasma cells	2297	0.29
Plasmablasts	2017	0.26
B-cells	1944	0.25
CD4+PD1+ T-cells	1815	0.23
Neutrophils	1754	0.22
CD4+CD8+ T-cells	1489	0.19
cDC1s	1110	0.14
NK T-cells	1069	0.14
CD4+Foxp3+ T-cells	984	0.13
CD8+ intraepithelial T-cells	901	0.11
CD8+ICOS+ T-cell	838	0.11
pDCs	738	0.09
CD8+Foxp3+ T-cells	563	0.07
NK cells	550	0.07
CD8+PD1+ T-cells	508	0.06
cDC2s	393	0.05
CD8+ICOS+PD1+ T-cells	212	0.03
CD4+ intraepithelial T-cells	149	0.02

RAR

Class_label	Count	Percentage
General unclassified	158672	21.89
Inflamed tubules	96113	13.26
Proximal tubules	63629	8.78
Distal collecting tubules	62211	8.58
Endothelial cells	60877	8.4
CD14+ Mφs	41138	5.67
HLAII- monocytes	33735	4.65
CD3+ T-cells	31090	4.29
HLAII+ monocytes	29348	4.05
CD8+ T-cells	22508	3.1
CD14+CD163+ Mφs	18813	2.6
CD14+MERTK+ Mφs	17802	2.46
CD4+ T-cells	16064	2.22
CD4+ICOS+PD1+ T-cells	7954	1.1
TCRγδ T-cells	6804	0.94
CD16+ Mφs	6310	0.87
CD14+CD163+MERTK+ Mφs	6291	0.87
CD4+ICOS+ T-cells	6237	0.86
CD4+CD8+ T-cells	4948	0.68
CD4+PD1+ T-cells	4491	0.62
B-cells	3774	0.52
CD8+ intraepithelial T-cells	3208	0.44
CD8+Foxp3+ T-cells	3104	0.43
Neutrophils	2864	0.4
NK T-cells	2638	0.36
CD4+Foxp3+ T-cells	2348	0.32
CD8+PD1+ T-cells	1734	0.24
CD8+ICOS+ T-cells	1680	0.23
Monocyte-derived Mφs	1662	0.23
CD8+ICOS+PD1+ T-cells	1174	0.16
NK cells	1139	0.16
Plasma cells	971	0.13
cDC1s	857	0.12
CD4+ intraepithelial T-cells	726	0.1
Plasmablasts	644	0.09
pDCs	628	0.09
cDC2s	341	0.05
RBCs	222	0.03

Casella, et. al, Supplemental Fig. 5

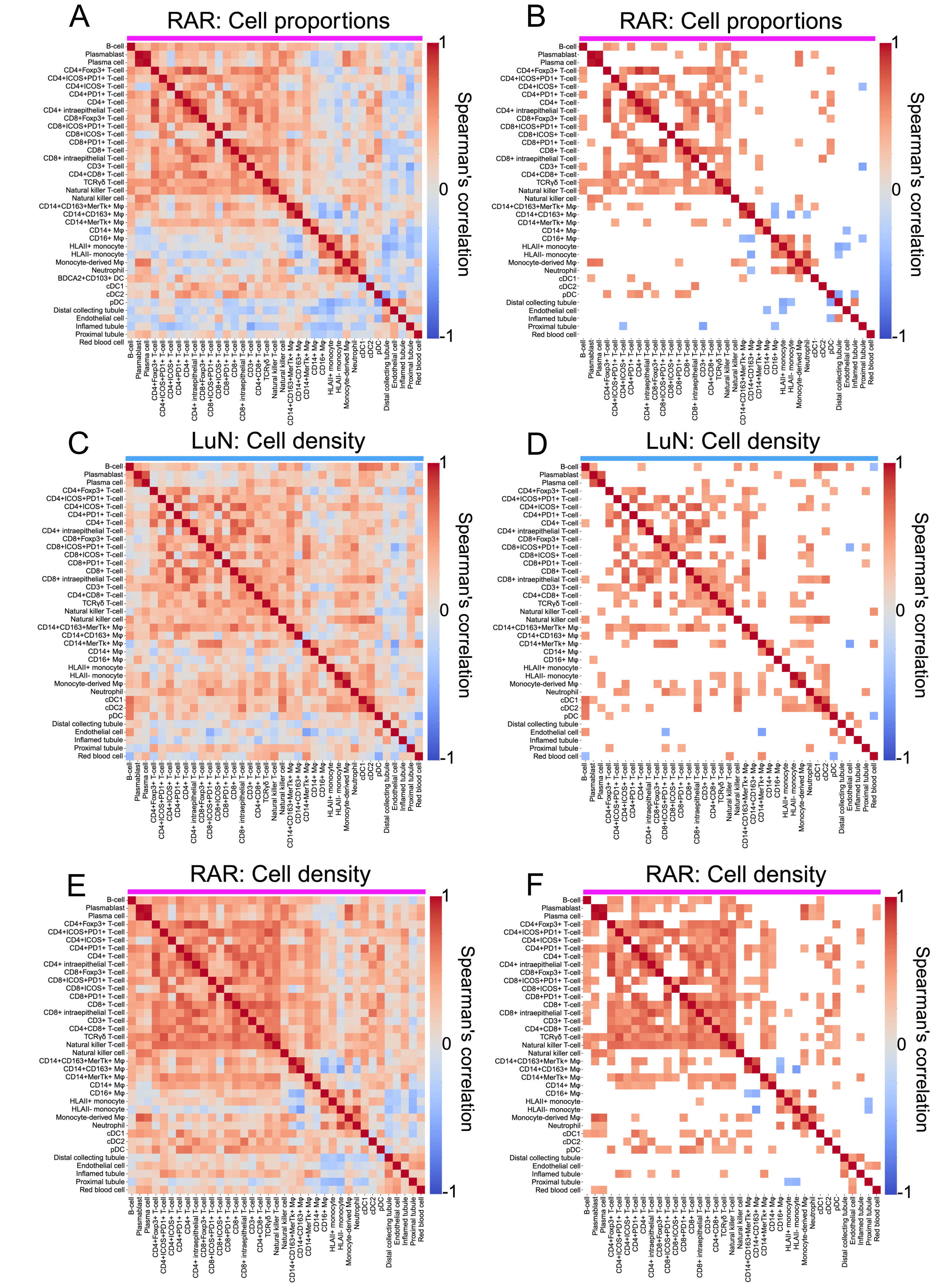

Casella, et. al, Supplemental Fig. 6

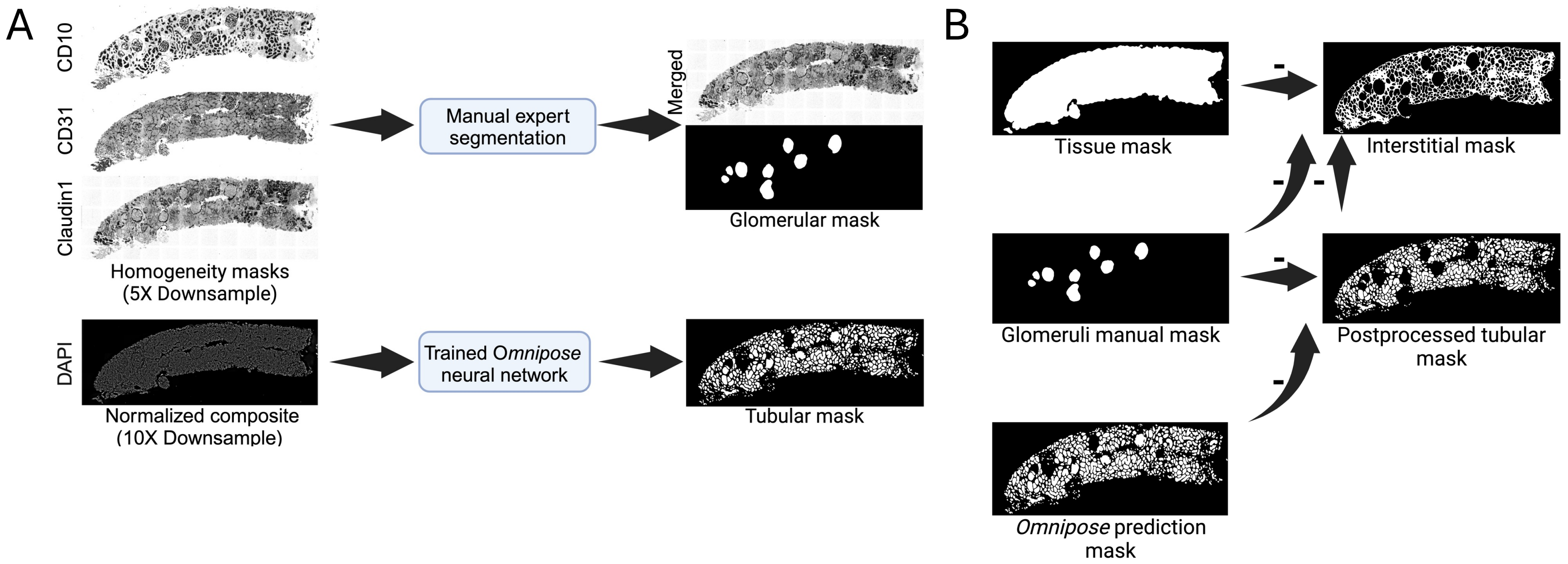

Casella, et. al, Supplemental Fig. 7

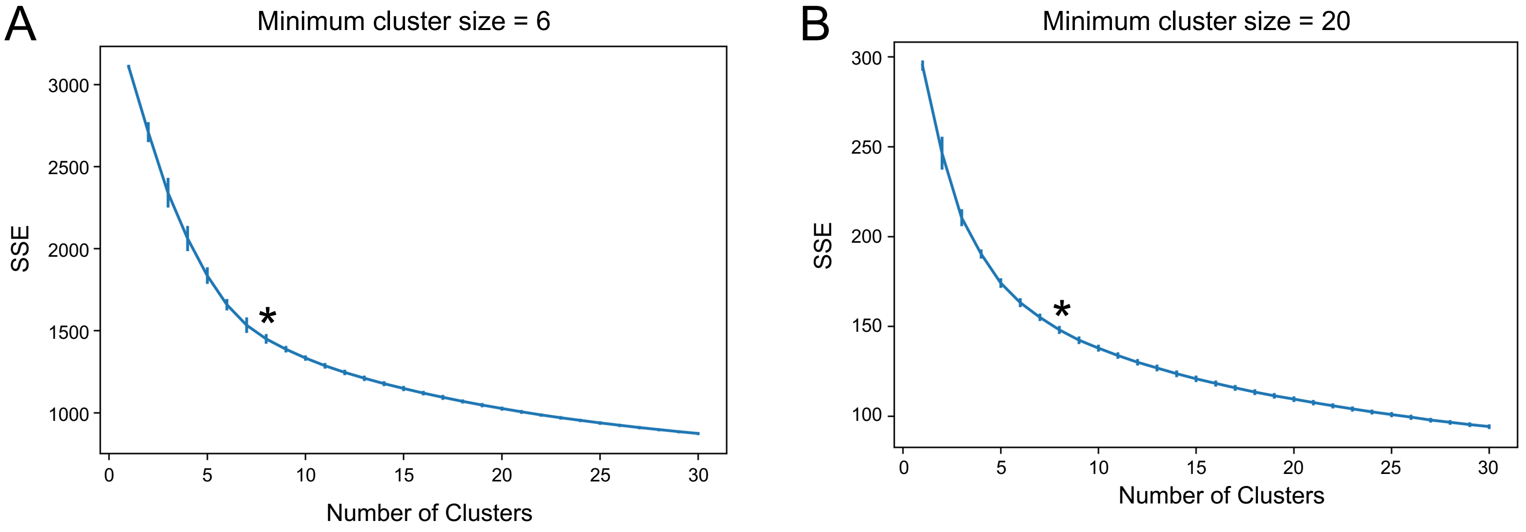

Casella, et. al, Supplemental Fig. 8

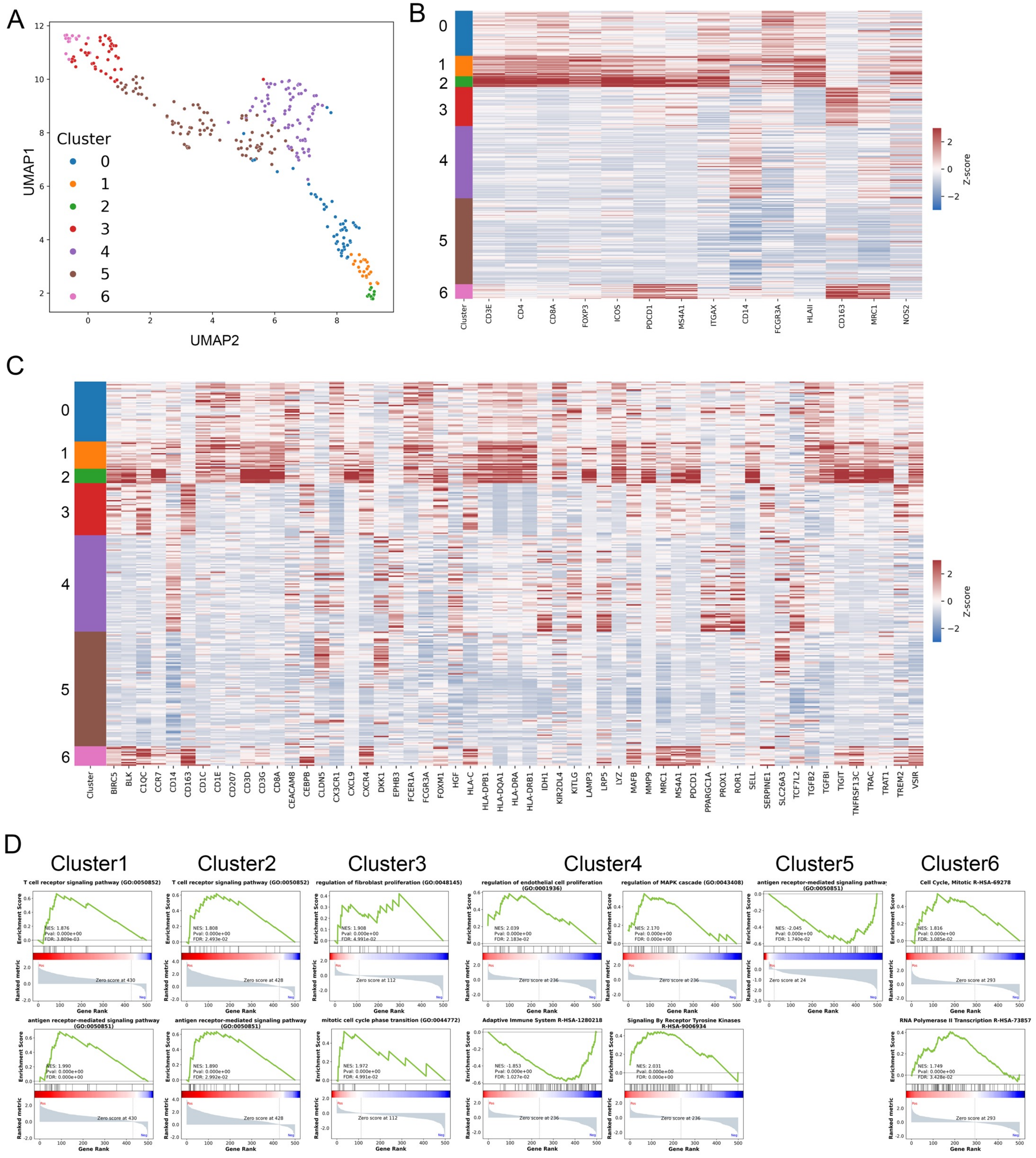

Casella, et. al, Supplemental Fig. 9

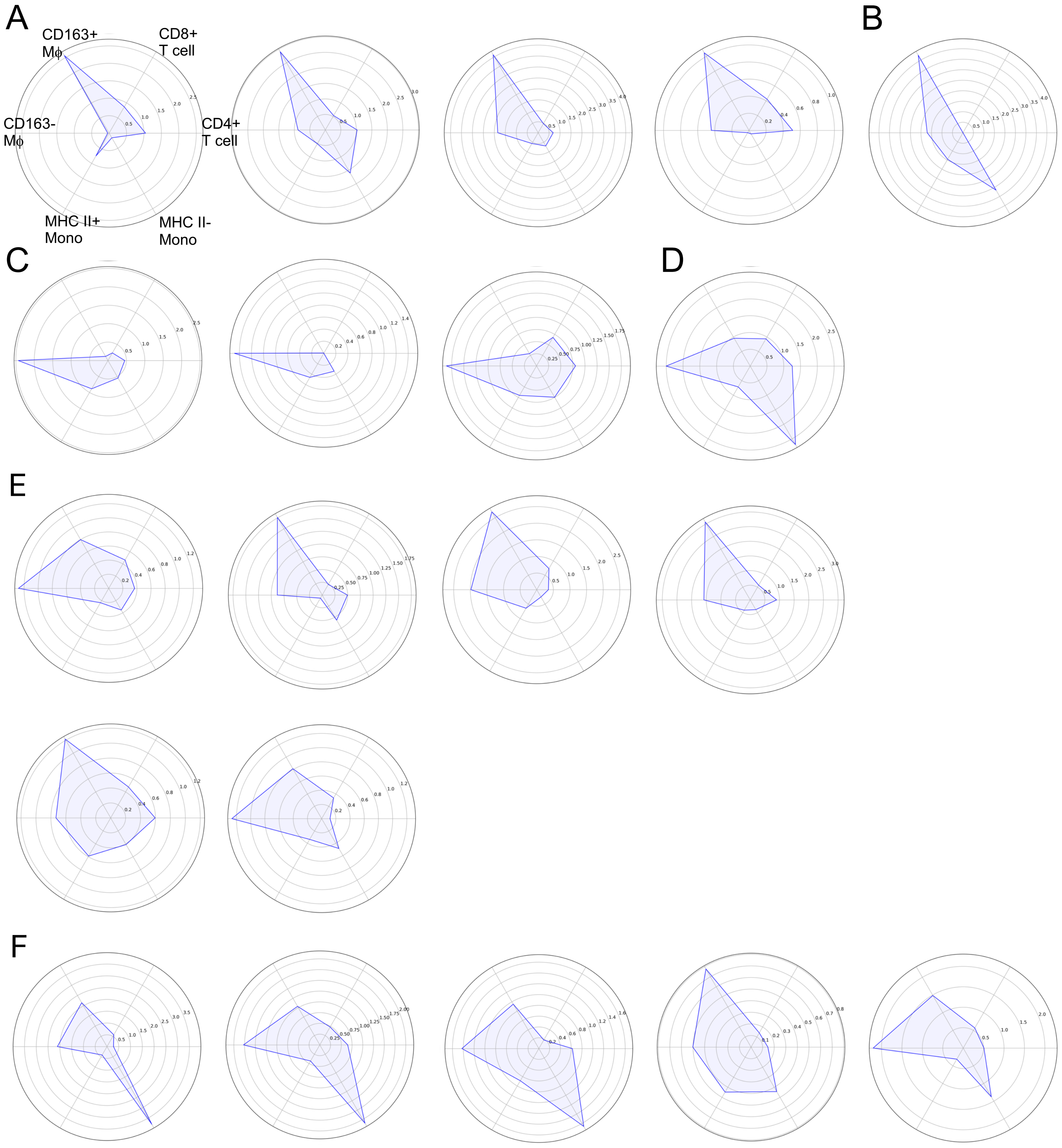

Casella, et. al, Supplemental Fig. 10

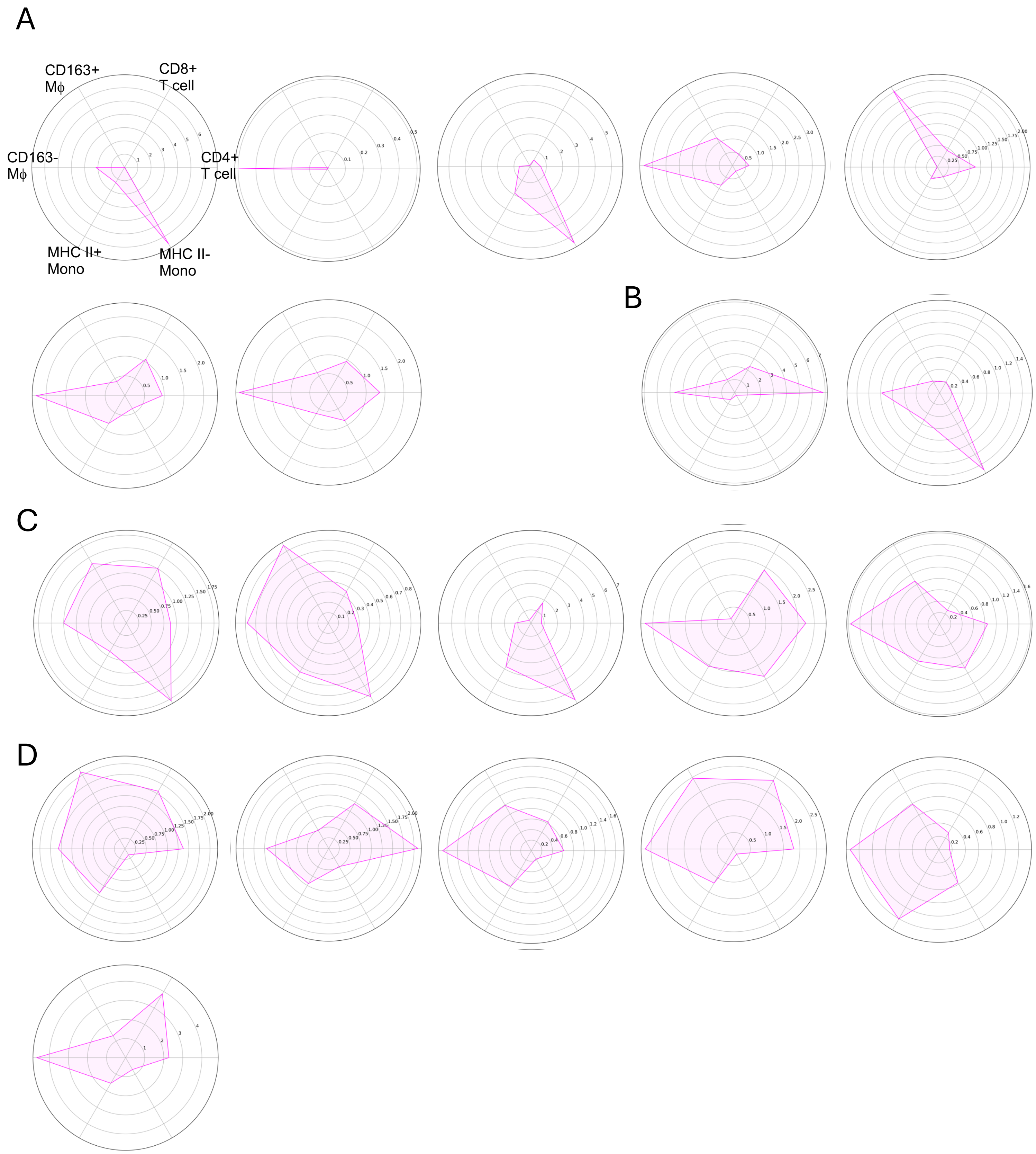

Casella, et. al, Supplemental Fig. 11

Supplemental Figure 1. Cohort demographics and Cellpose HITL retraining

A. Summary table of patient descriptors and characteristics for lupus nephritis patients. Includes ISN/RPS nephritis class 2-5.

B. Summary table of patient descriptors and characteristics for renal allograft rejection patients. Includes numbers with T-cell mediated rejection and mixed rejection. Other types of allograft rejection, such as antibody-mediated rejection, were excluded.

C. Cellpose F1-score performance before Human-in-the-loop retraining (solid line) and after HITL retraining (dashed line). Lupus nephritis is shown in blue. Renal allograft rejection is shown in magenta.

D. Cellpose Average Precision performance before Human-In-The-Loop retraining (solid line) and after HITL retraining (dashed line). Lupus nephritis is shown in blue. Renal Allograft Rejection is shown in magenta.

Supplemental Figure 2. Average MFI expression of the 42-marker panel.

A. UMAP dimensional reduction of cell body MFI from 30,000 cells randomly sampled. 10,000 cells are sampled from each of the three cohorts: KC, LuN, RAR. Shown are markers used for assigning cell class. MFI color scale is shown.

B. UMAP dimensional reduction of cell body MFI as described prior. Shown are markers not used for cell class assignment.

Supplemental Figure 3. Decision trees for cell class assignment.

A. Flow-cytometry analogous decision tree gating for immune cell classification based on cell body MFI.

B. Flow-cytometry analogous decision tree gating for non-immune cell, and CD45 low/negative myeloid cell, classification based on cell body MFI.

C. Decision tree gating for CD14+ macrophages (*left*) and monocytes (*right*).

D. Decision tree gating for CD8+ T-cells (*left*) and decision tree gating for CD4+ T-cells (*bottom right*).

Supplemental Figure 4. Cell classification of Cellpose segmented cells.

A. Summary table of the 33 immune and 5 non-immune cell classes ultimately assigned using the decision tree algorithm; cells are grouped by lineage.

B. Heatmap of the leave-one-out Z score (current cell class vs. all others) of the cell body MFI for the cell markers used in cell class assignment for the main immune and non-immune cell classes.

C. Heatmap of the cohort leave-one-out Z score (current cell class vs. all others) of the cell body MFI for the cell markers used in cell class assignment. Heatmaps for kidney control (*left*), lupus nephritis (*middle*), and renal allograft rejection (*right*).

D. Examples of normal, non-inflamed kidney tubules (left) and inflamed tubules expressing Claudin and MXA surrounded by COLIII fibrosis (right).

Supplemental Figure 5. Cell class frequency data.

A. Summary table of cell class total count and total percentage by disease cohort.

Supplemental Figure 6. Cell Class Density and Proportion Correlations

A. Heatmap of the non-parametric spearman's correlations between patient-level immune cell and non-immune cell proportions for renal allograft patients.
B. Heatmap of the non-parametric spearman's correlations between patient-level immune cell and non-immune cell proportions for renal allograft patients. Only significant correlations ($p<0.05$) are shown.
C. Heatmap of the non-parametric spearman's correlations between patient-level immune cell and non-immune cell density for lupus nephritis patients.
D. Heatmap of the non-parametric spearman's correlations between patient-level immune cell and non-immune cell density for lupus nephritis patients. Only significant correlations ($p<0.05$) are shown.
E. Heatmap of the non-parametric spearman's correlations between patient-level immune cell and non-immune cell density for renal allograft patients.
F. Heatmap of the non-parametric spearman's correlations between patient-level immune cell and non-immune cell density for renal allograft patients. Only significant correlations ($p<0.05$) are shown.

Supplemental Figure 7. Kidney compartment segmentation workflow.

A. Workflow of the procedure adopted for acquiring the computational segmentation of tubules and glomeruli.
B. Procedure adopted for the creation of the interstitial mask and post-processed tubular mask.

Supplemental Figure 8. **Estimation of the optimal cluster number.** DBSCAN cell networks Bootstrap estimate of optimal K Clusters DBSCAN cell networks using: no doublets, with doublets, and above 5 cell members, and above 19 cell members respectively. Points are the average of 3000 repetitions with corresponding error bars. (Bottom) Δ sum of squared distances, and (top) sum of squared distances is shown. Asterisk indicates heuristic K optimal clusters for respective bootstrap experiment.

Supplemental Figure 9. Cluster analysis using spatial transcriptomics (MERSCOPE) confirms CODEX clusters.

A. UMAP for labeled masks with K-mean clustering (k=7).

B. Heatmap of the seven clusters based on the selected genes corresponding to CODEX markers (Z-scores).

C. Heatmap of the distribution of the ten most highly differentially regulated genes in each cluster (55 genes total, Z-scores plotted).

D. Enrichment plots for statistically significant gene sets in each K-mean cluster vs. the other six clusters. False Discovery Rate (FDR) < 0.05.

Supplemental Figure 10. Principal immune cell trajectories of LuN biopsies.

A. Examples of individual LuN biopsies with predominant CD163+ macrophage trajectories.

B. Example of LuN biopsy with predominant CD163+ macrophage and HLA II- inflammatory monocyte trajectories.

C. Examples of LuN biopsies with predominant CD163- macrophage trajectories.

D. Example of LuN biopsy with predominant CD163- macrophage and HLA II- inflammatory monocyte trajectories.

E. Examples of LuN biopsies with both CD163+ and CD163- macrophage trajectories.

F. Examples of LuN biopsies with CD163+ and CD163- macrophage trajectories as well as HLA II- inflammatory monocyte trajectories.

Supplemental Figure 11. Principal immune cell trajectories of RAR biopsies.

A. Examples of RAR biopsies with predominance of a single immune cell trajectory.

B. Examples of RAR biopsies with two immune cell trajectories.

C. Examples of RAR biopsies with multiple immune cell trajectories including HLA II- inflammatory monocytes.

D. Examples of RAR biopsies with multiple immune cell trajectories excluding HLA II- inflammatory monocytes.
